# Effect of leukocyte and platelet-rich fibrin on free gingival graft healing: A clinical and histological study in rabbits

**DOI:** 10.34172/japid.2022.011

**Published:** 2022-08-06

**Authors:** Ahmad Mogharehabed, Nakisa Torabinia, Sare Sharifi Darani, Zohreh Afshari, Jaber Yaghini

**Affiliations:** ^1^Department of Periodontics, Dental Implants Research Center, Dental Research Institute, School of Dentistry, Isfahan University of Medical Sciences, Isfahan, Iran; ^2^Department of Oral and Maxillofacial Pathology, Dental Materials Research Center, Dental Research Institute, School of Dentistry, Isfahan University of Medical Sciences, Isfahan, Iran; ^3^Periodontist, Private Practice, Isfahan, Iran

**Keywords:** L-PRF, tissue grafts, wound healing

## Abstract

**Background.** Recently, the use of leukocyte- and platelet-rich fibrin (L-PRF) has been recommended due to the presence of various growth factors to increase the success of free gingival grafts (FGG). This study evaluated the effect of using L-PRF in the healing of FGG in rabbits.

**Methods.** Twenty rabbits were randomly divided into two groups. In each group, FGG was performed in two separate sites with or without L-PRF. One of these groups was sacrificed on the 7th day and the other on the 28th day and analyzed in terms of clinical indices, including wound healing, gingi­val thickness (GT), and keratinized tissue width (KTW). Then histologic sections were obtained and stained for type and degree of inflammation and rate of vascular formation analysis. SPSS 22 was used for statistical analysis.

**Results.** The extent of changes in GT, KTW, wound healing index, and vascular formation between the test and control groups was not statistically significant. The difference in the type of inflammation was significant only between the -7day and -28day control groups (P=0.003). The degree of inflammation between the -7day test group and the -28day control group, as well as the -7day and -28day control groups, were statistically significant (P=0.011 and P=0.002, respectively).

**Conclusion.** Using L-PRF with FGG could improve FGG healing compared to using FGG alone, but the results were not statistically significant.

## Introduction

 Free gingival autograft (FGG) is a simple, easy, and highly predictable gingival augmentation modality. The free gingival autograft was first described by Bjorn in 1963 and Sullivan & Atkins in 1968.^1,2^ In this method, the placement of a keratinized tissue band, although narrow, is expected. Today, the gold standard to augment keratinized gingiva is an FGG harvested from the palate.^3^

 Nowadays, the interest in using growth factors to increase the speed of the healing process through natural biological pathways has increased. A new technique for the use of concentrated platelets is the use of L-PRF. L-PRF is a second-generation platelet concentrate introduced by Choukroun et al in 2001.^4^ Its three-dimensional fibrin network promotes neovascularization and accelerates wound closing and fast cicatricial tissue remodeling.^5^

 Platelet concentrates are a source of autologous growth factors that promote cell migration and proliferation. Given that L-PRF is produced without using any additives, the fibrin polymerization occurs physiologically, resulting in a similar fibrin network as the one formed during natural healing. It contains many growth factors and cytokines, including vascular endothelial growth factor (VEGF), epidermal growth factor, transforming growth factor-β1, interleukin-1β (IL-1β), IL-4, IL-6, and tumor necrosis factor-α. These factors are believed to be beneficial for wound healing.^5,6^

 Recent evidence on the use of L-PRF in oral applications suggests that it improves soft tissue wound healing, periodontal regeneration, and healing of extraction sockets.^7-9^ In most of these procedures, L-PRF is placed below oral mucosal gingival flaps.

 Meza-Mauricio et al^10^ conducted a systematic review of randomized clinical studies in 2021 and showed that the PRF membrane improved wound healing during the first two weeks and promoted less postoperative pain. In another systematic review in 2021, Mancini et al^11^ studied the efficacy of L-PRF in addition to coronally advanced flap (CAF) for the treatment of both single and multiple gingival recessions compared to the CAF alone and the adjunct connective tissue graft (CTG). No statistically significant differences were found between the two groups regarding KTW gain, but CAF + L-PRF achieved significantly greater GT gain than CAF alone.

 The healing of FGG is strongly influenced by angiogenesis; on the other hand, L-PRF induces angiogenesis.^12,13^ Therefore, the researchers suggested that using L-PRF under FGG may accelerate the healing of gingival grafts. Since no study has evaluated this subject, this study aimed to assess the clinical and histological effects of the L-PRF membranes in FGG healing in rabbits.

## Methods

 Twenty male New Zealand white rabbits with an average weight of 1.5 kg were used in this study. Inclusion criteria consisted of the similarity of rabbits in terms of age, sex, breed, diet, and the housing environment, and exclusion criteria included the presence of disease and differences in age, sex, and breed. Considering a type 1 error of 5% and 80% power, the sample size was calculated at n=10 in each group.

 The animals were randomly divided into two groups: experimental (L-PRF + FGG) and control (FGG). Randomization was performed based on a table of random numbers.

###  Surgical and experimental protocol 

 To anesthetize the rabbits, 10% ketamine hydrochloride (Alfasan, Woerden, Netherlands) at a dose of 30 mg⁄kg and 2% xylazine (Alfasan, Woerden, Netherlands) at a dose of 3 mg⁄kg were injected intramuscularly in the upper outer quarter of the thigh quadriceps muscle of the animals.

1. Preparation of L-PRF: A standard cardiac venipuncture was performed. Blood was drawn into four 9-mL tubes without anticoagulant. L-PRF clots and membranes were prepared as described by Choukroun et al.^4^ The tubes were immediately centrifuged at 2700 rpm (408 g) for 12 minutes. After centrifugation, each L-PRF clot was removed from the tube and separated from the red element phase at the base with pliers (Figure 1). L-PRF clots were gently squeezed between a sterile glass plate and a metal box (gravity, no loading).
  **Figure 1:**
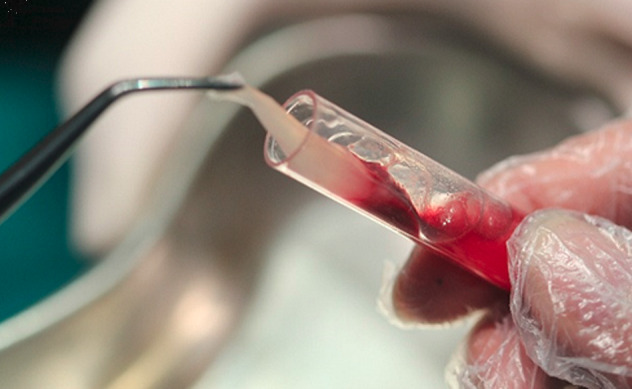
Preparation of L-PRF according to Choukroun et al.
2. At the recipient site, a superficial, 4-mm, split-thickness flap preparation was performed using a 15C blade towards the vestibular side, and releasing incisions were performed.^14^
3. FGG was made available from the palatal tissue (Figure 2. A.)
  **Figure 2:**
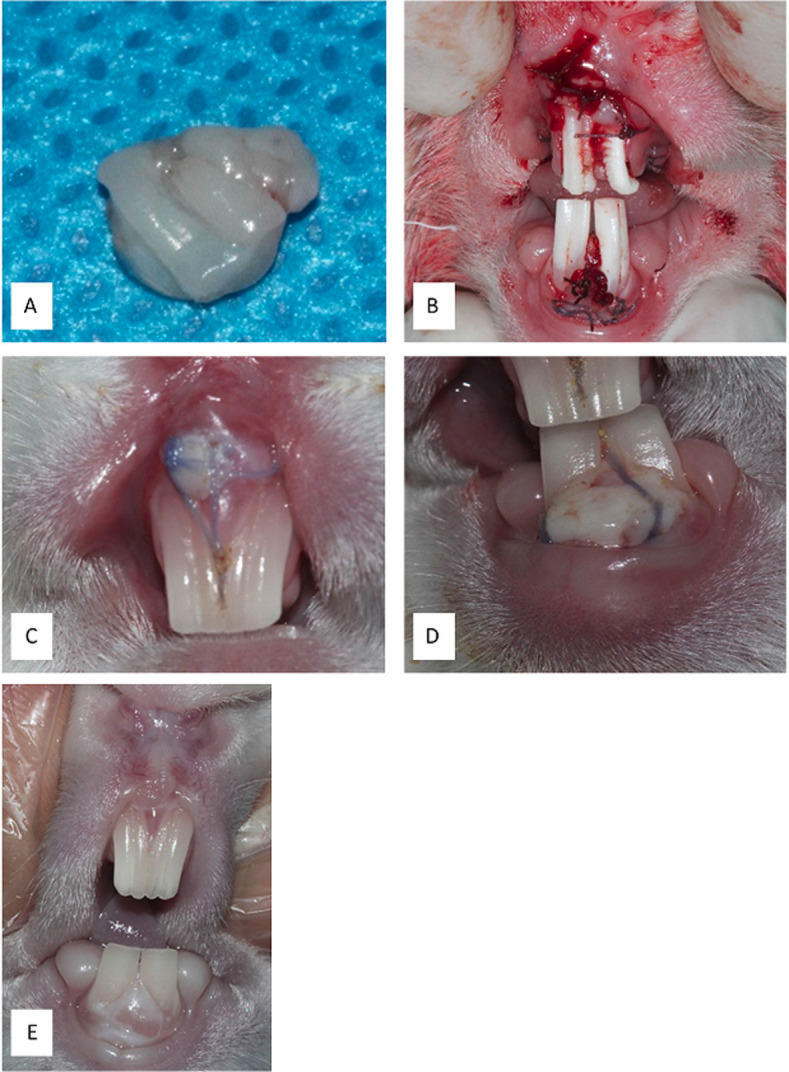
A) FGG was made available from palatal tissue. B) FGG with and without L-PRF and suturing. C) 7-day follow-up of FGG with L-PRF in the anterior maxilla. D) 7-day follow-up of FGG without L-PRF in the anterior mandible. E) 28-day follow-up of anterior maxilla and mandible.
4. L-PRF membrane and FGG were trimmed according to graft site and sutured with resorbable sutures (Vycril 4.0, Ethicon®). First, L-PRF was sutured on the graft site; then, FGG was sutured on it (Figure 2. B. maxilla).
5. In control sites, the same surgical protocol was followed except for L-PRF use (Figure 2. B. mandible).
6. After surgery, each animal was given penicillin intramuscularly at a dose of 60000 mg/kg. All the sutures were removed after 7 days.

Histological analyses were carried out 7 and 28 days after FGG. At each time interval of analysis (days 7 and 28), 10 animals, 5 per group, were killed by an overdose of ketamine and xylazine. GT and KTW were evaluated at baseline and 7 and 28 days after surgery by an endodontic reamer with a silicon stop.^15^ Furthermore, the wound healing index was assessed 7 and 28 days after surgery.

 The wound healing index was graded as below: Grade 1: healing without any major events, with no edema, gingival erythema, pus discharge, or opening of the wound edges. Grade 2: healing without any major events; healing with a small amount of edema or gingival erythema or opening of the wound edges, but without any discharge of pus. Grade 3: poor wound healing with marked gingival edema, erythema, and opening of the wound edges or any pus discharge.^16^

 All the surgical procedures were performed by an experienced surgeon (A.M.), and all the measurements were made by another blinded operator (S.Sh.).

###  Specimen preparation

 A 15C blade was used to obtain the full-thickness samples. Each sample was placed separately in a container with 10% formalin. The samples were prepared by hematoxylin and eosin staining (H&E) (for type and degree of inflammation,^17^ epithelium, fibrous tissue, fibrin, and granulation tissue formation analysis^18^) and a VEGF marker (new vessel assessment^19^). The slides were examined under a light microscope. Donor sites were prepared by H&E staining. The pathologist was blinded to the two groups (N.T.).

###  Statistical analysis

 Statistical analyses were performed using SPSS at a significance level of 5%. The data were analyzed using Kruskal-Wallis, Wilcoxon, Mann-Whitney, and Fisher’s exact tests.

## Results

 In this animal study, 20 adult male New Zealand rabbits were randomly divided into two groups. In each case, FGG was performed in the anterior maxillary and mandibular jaws with or without L-PRF. In the palatal area, the wound was covered with L-PRF or without it. All the animals recovered from surgery without complications. The clinical indices were evaluated, tissue samples were prepared for histological examination, and statistical analyses were performed.

###  Recipient site results

 Changes in the GT and KTW, differences in wound healing index, and the incidence of VEGF at the beginning and end of the study were not statistically significant between the four groups.

 One week after surgery, changes in KTW in the test group were statistically significant (P=0.014) compared to the baseline but not significant in the control group (P=0.131). After 28 days, changes in KTW were significant between the test and control groups (P=0.016 and P=0.014, respectively). After 7 and 28 days, the differences in GT were statistically significant between the test and control groups compared to the baseline.

 After 7 days, the mean vascular formation was 37.5% in the test group, with 39.3% in the control group. These values ​​were 33% and 31.1% in the test and control groups, respectively, after 28 days.

 Kruskal-Wallis test showed significant differences in inflammation severity between the four groups (P=0.001). Therefore, the Mann-Whitney test was used for two-by-two comparisons of the groups. The differences in inflammation severity were statistically significant only between the control groups on the 7th day and the control groups on the 28th day (P=0.003).

 The differences in inflammation severity were significant between the four groups (P=0.003). On the 7th day, two cases of grade 1 and eight cases of grade 2 inflammation were recorded in the test group, and one case of grade 1, seven cases of grade 2, and two cases of grade 3 inflammation were recorded in the control group. None of the samples had grade 3 inflammation. One sample from the test group and three samples from the control group had no inflammation.

**Figure 3:**
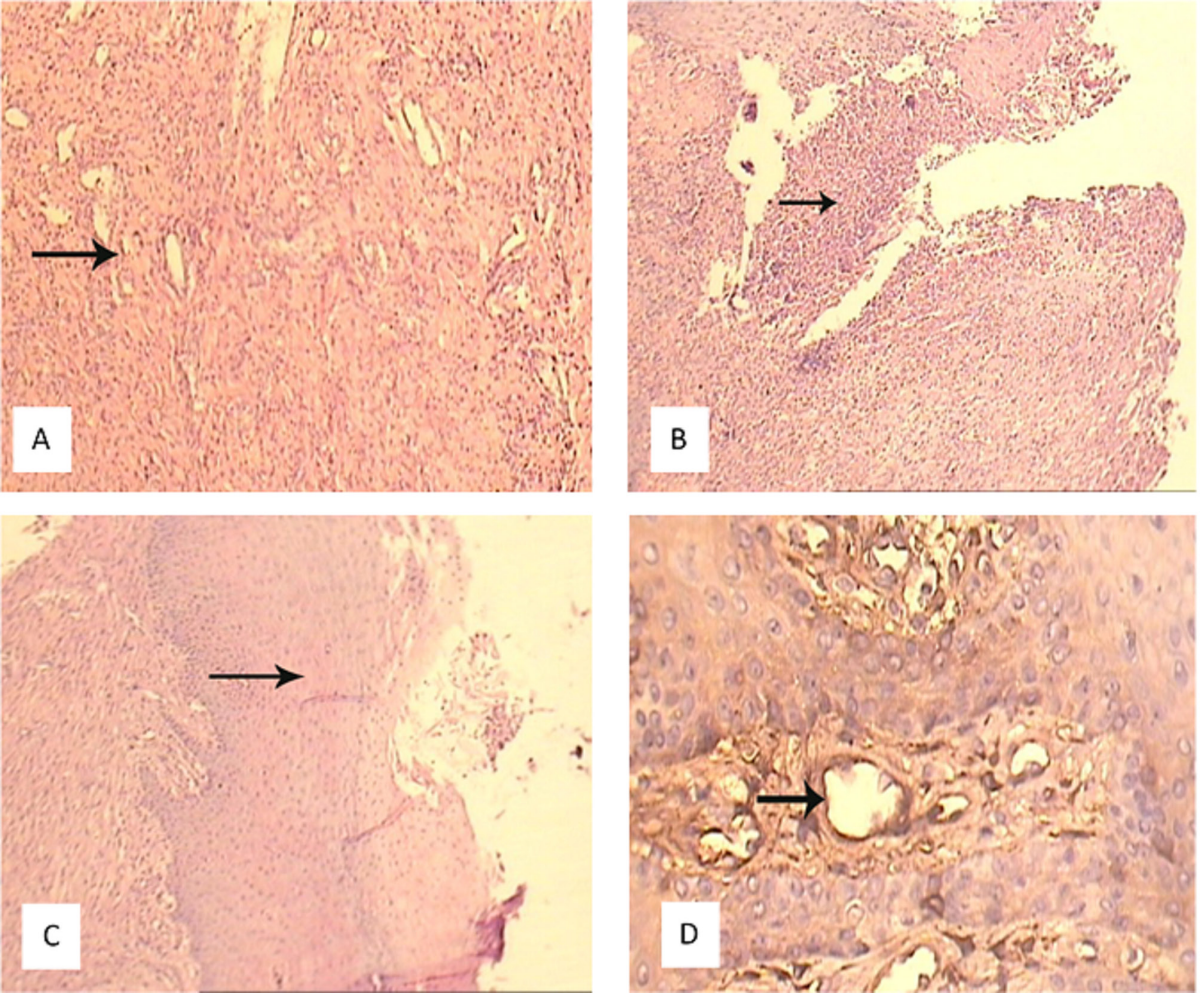
A) Granulation tissue *10. B) Fibrin *10. C) Epithelium *10. D) Vessels *40.

 Fisher’s exact test showed a statistically significant difference between the four groups regarding fibrin formation and fibrous tissue in the histological analysis. However, these differences ​​were not significant regarding granulation tissue and epithelium (Figure 3, Table 1).

**Table 1:** Frequency distributions of measured parameters in the recipient site

	**7th day**		**28th day**		
	**Control**	**Test**	**Control**	**Test**	
**KTW**					**P-value**
**Baseline**	4.4±0.69	4±0.47	4.2±0.63	4.31±0.67	0.444
**28th day**	4.85±0.81	4.6±0.51	5.1±0.56	5.4±0.51	0.052
**Changes**	0.45±0.89	0.6±0.51	0.9±0.73	1.1±0.99	0.402
**GT**					**P-value**
**Baseline**	1.85±0.24	1.8±0.25	1.9±0.39	2±0.66	0.585
**28th day**	2.6±0.61	2.8±0.67	2.45±0.59	2.85±0.33	0.320
**Changes**	0.55±0.48	1±0.52	0.55±0.49	0.85±0.66	0.407
**Wound healing index**					
**1**	5	8	8	10	
**2**	5	2	2	0	
**3**	0	0	0	0	
**Vessel formation**					
**mean**	39.3	37.5	31.1	33	
**Degree of inflammation**					
**normal**	0	0	3	1	
**+**	1	2	5	2	
**++**	7	8	2	7	
**+++**	2	0	0	0	
**mean**	2.1	1.8	0.9	1.6	
**P-value**					
**Fibrin formation**	8	7	0	2	0.001
**Granulation tissue**	4	3	5	6	0.715
**Fibrous tissue**	1	2	7	3	0.043
**Epithelium**	0	0	4	1	0.64

###  Donor sites results

 The difference in wound healing index between the four groups (P=0.001) and between the 7-day study group and the 7-day control group was significant (P=0.007). On the 7th day, all the test groups showed grade 2, and in the control group, 3 cases of grade 3 and 7 cases of grade 2 were detected. In the 28-day evaluation, all cases in the experimental group and 6 cases in the control group showed first-degree wound healing, and four cases in the control group showed second degree.

 The difference in the type of inflammation between the four groups was significant (P=0.009). In addition, the difference in inflammation severity between the 28-day test group and the 7-day control group was statistically significant (P=0.023).

 In the test group, after 28 days, all the samples were in a non-inflammatory condition, while in the control group, one sample had chronic inflammation, and the rest were normal. Also, after seven days, no inflammation was observed in eight samples of the experimental group and four samples of the control group.

 The difference in the degree of inflammation between the four groups was significant (P=0.012). In addition, this difference between the 28-day study group and the 7-day control group and between the 7-day control group and the 28-day control group was significant (P=0.023 and P=0.043, respectively).

 Examination of fibrin, granulation tissue, fibrous tissue, and epithelium in histological samples did not show a statistically significant difference between the four groups (Table 2).

**Table 2:** Frequency distributions of measured parameters in the donor site

	**7th day**		**28th day**		
	**Control**	**Test**	**Control**	**Test**	
**Wound healing index**					
**1**	3	10	6	10	
**2**	7	0	4	0	
**3**	0	0	0	0	
**Degree of inflammation**					
**Normal**	4	8	9	10	
**+**	3	0	1	0	
**++**	3	1	0	0	
**+++**	0	1	0	0	
**Mean**	2.2	0.5	2.9	0	
**P-value**					
**Fibrin formation**	2	2	0	0	0.3
**Granulation tissue**	1	1	0	0	0.99
**Fibrous tissue**	7	1	4	4	0.065
**Epithelium**	6	9	8	10	0.163

## Discussion

 The use of L-PRF membranes has also become common in periodontal surgery due to its accelerating healing properties and the presence of anti-inflammatory growth factors and cytokines. Therefore, this study aimed to evaluate clinical indicators such as changes in KTW and GT and wound healing index and histological evaluation of vascular formation percentage, type, and severity of inflammation and tissue repair process using L-PRF under FGG in an animal model.

 This study showed that the difference in the KTW at baseline between the four groups was not significant, indicating the same conditions for these four groups. The use of L-PRF with FGG could not have a significant effect in terms of increasing the KTW compared to FGG alone. According to histological findings, there was no significant improvement in histological repair in the L-PRF group compared to the control group, which could improve clinical indicators, including KTW. Amr et al^20^ showed that using platelet-rich fibrin beneath FGG in gingival augmentation surgery resulted in a successful increase in the KTW compared to using FGG alone.

 In this study, GT showed more difference between the control and study groups compared to other indices, although this difference was not statistically significant. Two mechanisms have been mentioned to increase GT after L-PRF application: 1) the space created by L-PRF; 2) L-PRF regulates the proliferation of specific cells such as periodontal ligament cells and gingival fibroblasts and epithelial cells.^21-24^ In the current study, since granulation tissue and epithelial formation did not show any histological differences between the test and control groups, the researchers believe that the cause of the increase in GT was the larger space created by L-PRF than its stimulatory effects on growth factors.

 In this study, the difference in wound healing index, which is a clinical indicator, was not significant between the test and control groups. This means that using L-PRF, based on wound healing indicators, could not accelerate the healing of FGG, indicating that L-PRF could regulate the expression of genes related to early wound healing in human gingival fibroblasts.^6^ This can be the reason for the acceleration of wound healing with L-PRF, which has been seen in other intraoral surgeries, including tooth extraction.^7,9^ This index is an observer-dependent index, and different indicators have been used in these studies. Perhaps these reasons can explain the differences in the results. However, the design and conditions of the study can also be effective.

 What distinguishes this study from similar studies is histological and clinical examinations that can complement each other and justify the results. In this study, in the histological examination of the samples, the formation or absence of granulation tissue and epithelium was not statistically significant. The formation and no formation of fibrin were different only between the 7-day test group and 28-day control groups, the 28-day test group and the 7-day control groups, and the control groups. The formation and no formation of fibrous tissue were different only between the control groups. Also, the percentage of vascular formation did not significantly differ between the four groups.

 Three important factors are required for wound healing and maturation, including vascular formation, immune responses and control of circulating stem cells, and wound protection by the epithelium.^5^ One of the important components in L-PRF is VEGF. It is the most important growth factor in vascular formation. In the present study, the rate of neovascularization in the test and control groups was not significantly different, and L-PRF could not increase its rate. It depends on the removal of inflammatory cells from the site and the transfer of reparative cells to the site, and this operation is performed by blood vessels. Blood vessels and blood circulation have a key role in the healing process and determine the next clinical events. Therefore, researchers believe that the similar clinical process in the wound healing process in the test and control groups is mainly because the rate of neovascularization is not different in the two groups, and the effect of L-PRF, in this case, was not obvious.

 Regarding the severity of inflammation, there was only a statistically significant difference between the 7-day and 28-day control groups. The 7-day control group showed more acute inflammation, and the 28-day control group showed more chronic inflammation. Although this difference was not significant in the study groups, the fact is that in the 7-day study group, there was more acute inflammation, and in the 28-day study group there was more chronic inflammation, which shows the normal process of graft healing in the study groups and the transition from acute inflammation to the chronic inflammation and ultimately tissue healing and maturation. The degree of inflammation differed between the 28-day study group and its control group, as well as the 7-day and 28-day control groups. There are no similar studies on the severity and type of inflammation in gingival grafts with L-PRF.

 In this study, the wound healing process in the donor area was also examined. Placement of L-PRF in the soft tissue donor area stopped bleeding and helped rapid homeostasis. The difference in wound healing index between the 7-day test group and its control, as well as the 28-day test group and its control group, was significant, indicating that the positive effect of L-PRF served as a wound dressing and accelerated the migration of epithelial cells to cover the area. Meza-Mauricio^10^ (2021) in a review study, assessed the wound healing index after using a PRF membrane to protect the palatal donor site following free gingival graft harvesting procedures and reported that the PRF membrane accelerated wound healing. Also, Gusman et al^25^ showed in a systematic review that using PRF for wound healing of palatal donor sites of FGG might decrease postoperative pain and induce earlier complete wound epithelialization.

 Regarding the severity of inflammation, only the difference between the 28-day study group and its control group was significant. In the histological examination of the grafted tissue, no significant difference was detected in the formation or absence of epithelium, fibrin, fibrous tissue, and granulation tissue.

 Due to the small size of the rabbit and the consequent low blood volume, low blood pressure, and small blood vessels, rapid blood sampling was very difficult. In addition, it was impossible to re-sample each rabbit at a time. Therefore, blood sampling was carried out through the heart, which carried the risk of animal death. Dohan et al^26^ also encountered these problems in 2010 and recommended using larger animals such as dogs and goats. Another problem was the lack of a similar study using FGG with L-PRF and the impossibility of accurate comparison with similar samples.

## Conclusions

 It can be concluded from the results of this study that the use of L-PRF with FGG improved clinical indicators such as KTW, GT, and wound healing index. However, these differences were not supported by histological studies performed in this study. Although various studies have discussed the many benefits of L-PRF and several clinical effects have been predicted, only studies with accurate clinical evaluations and histological examinations can make good judgments about its effectiveness. Unfortunately, there are no sufficient studies of this type available at the moment. Therefore researchers suggest that since using L-PRF with gingival grafting requires additional costs and trauma to the patient, further studies are necessary to clarify the true clinical effects. However, it should be noted that the effects of L-PRF are more pronounced in areas where secondary healing is performed, such as areas that are protected after tooth extraction. In the present study, the positive effects of using the L-PRF in the donor area were much more significant than in the recipient area.

## Acknowledgments

 None.

## Competing Interests

 The authors declare no conflicts of interest.

## Authors’ Contributions

 AM: Conceptualization, methodology, acquisition, writing, review, and editing

 NT: Methodology, acquisition, histological analysis, interpretation, writing the original draft, reviewing and editing

 SSD: Formal analysis, interpretation, writing the original draft, writing, reviewing, and editing

 ZA: Formal analysis, interpretation, writing the original draft, writing, reviewing, and editing

 JY: Conceptualization, methodology, acquisition, writing, reviewing, and editing.

## Funding

 This study was supported by the Isfahan University of Medical Sciences.

## Availability of data

 Available.

## Ethics Approval

 All stages and methods of the study were approved by the Ethics Committee of Animal Studies of Isfahan University of Medical Sciences and approved with the code 3941018.
